# Oxalate-Degrading Bacillus subtilis Mitigates Urolithiasis in a Drosophila melanogaster Model

**DOI:** 10.1128/mSphere.00498-20

**Published:** 2020-09-09

**Authors:** Kait F. Al, Brendan A. Daisley, Ryan M. Chanyi, Jennifer Bjazevic, Hassan Razvi, Gregor Reid, Jeremy P. Burton

**Affiliations:** a Canadian Centre for Human Microbiome and Probiotic Research, Lawson Health Research Institute, London, Ontario, Canada; b Department of Microbiology and Immunology, Western University, London, Ontario, Canada; c Division of Urology, Department of Surgery, Western University, London, Ontario, Canada; University of California, Davis

**Keywords:** *Bacillus subtilis*, *Drosophila*, calcium oxalate, host-microbe interactions, microbiota, nephrolithiasis, probiotics

## Abstract

Kidney stones affect nearly 10% of the population in North America and are associated with high morbidity and recurrence, yet novel prevention strategies are lacking. Recent evidence suggests that the human gut microbiota can influence the development of nephrolithiasis, although clinical trials have been limited and inconclusive in determining the potential for microbially based interventions. Here, we used an established Drosophila melanogaster model of urolithiasis as a high-throughput screening platform for evaluation of the therapeutic potential of oxalate-degrading bacteria in calcium oxalate (CaOx) nephrolithiasis. The results demonstrated that Bacillus subtilis 168 (BS168) is a promising candidate based on its preferential growth in high oxalate concentrations, its ability to stably colonize the D. melanogaster intestinal tract for as long as 5 days, and its prevention of oxalate-induced microbiota dysbiosis. Single-dose BS168 supplementation exerted beneficial effects on D. melanogaster for as long as 14 days, decreasing stone burden in dissected Malpighian tubules and fecal excreta while increasing survival and behavioral markers of health over those of nonsupplemented lithogenic controls. These findings were complemented by *in vitro* experiments using the established MDCK renal cell line, which demonstrated that BS168 pretreatment prevented increased CaOx crystal adhesion and aggregation. Taking our results together, this study supports the notion that BS168 can functionally reduce CaOx stone burden *in vivo* through its capacity for oxalate degradation. Given the favorable safety profile of many B. subtilis strains already used as digestive aids and in fermented foods, these findings suggest that BS168 could represent a novel therapeutic adjunct to reduce the incidence of recurrent CaOx nephrolithiasis in high-risk patients.

**IMPORTANCE** Kidney stone disease is a morbid condition that is increasing in prevalence, with few nonsurgical treatment options. The majority of stones are composed of calcium oxalate. Unlike humans, some microbes can break down oxalate, suggesting that microbial therapeutics may provide a novel treatment for kidney stone patients. This study demonstrated that Bacillus subtilis 168 (BS168) decreased stone burden, improved health, and complemented the microbiota in a Drosophila melanogaster urolithiasis model, while not exacerbating calcium oxalate aggregation or adhesion to renal cells *in vitro*. These results identify this bacterium as a candidate for ameliorating stone formation; given that other strains of B. subtilis are components of fermented foods and are used as probiotics for digestive health, strain 168 warrants testing in humans. With the severe burden that recurrent kidney stone disease imposes on patients and the health care system, this microbial therapeutic approach could provide an inexpensive therapeutic adjunct.

## Abstract (precis)

Kidney stone disease is a morbid condition that is increasing in prevalence, with few nonsurgical treatment options. The majority of stones are composed of calcium oxalate. Unlike humans, some microbes can break down oxalate, suggesting that microbial therapeutics may provide a novel treatment for kidney stone patients. This study demonstrated that Bacillus subtilis 168 (BS168) decreased stone burden, improved health, and complemented the microbiota in a Drosophila melanogaster urolithiasis model, while not exacerbating calcium oxalate aggregation or adhesion to renal cells *in vitro*. These results identify this bacterium as a candidate for ameliorating stone formation; given that other strains of B. subtilis are components of fermented foods and are used as probiotics for digestive health, strain 168 warrants testing in humans. With the severe burden that recurrent kidney stone disease imposes on patients and the health care system, this microbial therapeutic approach could provide an inexpensive therapeutic adjunct.

## INTRODUCTION

Nephrolithiasis, or kidney stone disease, affects approximately 10% of North Americans and continues to increase in prevalence (1). The condition is associated with significant patient morbidity and poses a severe economic burden to health care systems, since many cases are recurrent and require surgical management (1). Unfortunately, novel prevention strategies for this disease are lacking.

Approximately 80% of all kidney stones are composed of calcium oxalate (CaOx), with oxalate and calcium excretion, urine volume, and the presence of crystallization inhibitors all playing pivotal roles in their formation (1). Urinary oxalate is derived from both dietary and endogenous sources, and estimates of the relative contributions vary widely; studies have suggested that the proportion of diet-derived urinary oxalate ranges from 10 to 50% (2–4). Typically, dietary oxalate ingestion is approximately 1 to 2 mM; it can be absorbed by both trans- and paracellular mechanisms in its soluble form, excreted in the feces as an insoluble crystal, or degraded by members of the gut microbiota (3, 5). The bacterium Oxalobacter formigenes utilizes oxalate as a carbon source via formyl-CoA transferase and oxalyl-CoA decarboxylase (EC 2.8.3.16 and EC 4.1.1.8, respectively), and when it is present in the intestine, people have been reported to have lower urinary oxalate levels and to be at lower risk of developing stones (6, 7). Other members of the gut microbial community, including lactobacilli and bifidobacteria, are capable of degrading oxalate, though to a lesser extent (8). For this reason, supplementation with oxalate-degrading bacteria in kidney stone patients has been suggested as a potential preventive therapy; however, trials thus far have been limited and inconclusive (9–13).

Like O. formigenes, Bacillus subtilis 168 (BS168) has been shown to degrade oxalate (14). The strain’s oxalate decarboxylase gene, *yvrK*, is acid induced and encodes a ∼43-kDa manganese-requiring enzyme (EC 4.1.1.2) that converts oxalate to formate and CO_2_ (15). A few *Bacillus* spp. have recently been identified for potential probiotic use for gastrointestinal disorders, with the added benefit of being highly heat, salt, and pH resistant due to spore formation (16, 17). The aim of the present study was to investigate strain 168 for its therapeutic potential in CaOx nephrolithiasis treatment.

A Drosophila melanogaster model of CaOx urolithiasis was utilized in conjunction with 16S rRNA gene sequencing and quantitative PCR (qPCR) to evaluate the effects of supplementation with BS168 on stone burden and the indigenous microbiota. In addition, MDCK renal epithelial cells were used to determine the impact of BS168 on CaOx crystal adhesion and aggregation. It was hypothesized that based on its oxalate-degrading ability, BS168 would reduce stone burden and promote markers of health.

## RESULTS

### Increasing oxalate concentration promotes the growth of BS168.

**FIG 1:**
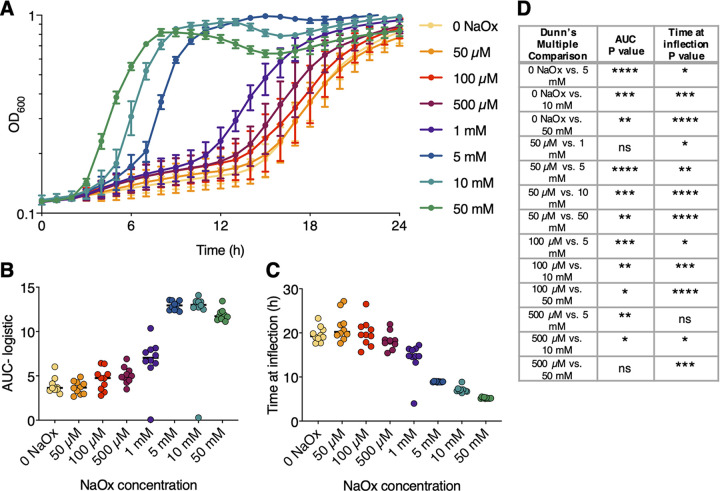
Increasing the concentration of oxalate promotes the growth of BS168. (A) Growth is represented as the increase in optical density at 600 nm over the course of 24 h. BS168 was grown in LB broth with NaOx at the indicated concentrations. Three biological replicates were performed; the means of 10 representative replicates and standard deviations are plotted. (B) Area under the logistic curve from panel A (*n *= 10). (C) Time at curve inflection from panel A (*n *= 10). (D) Comparisons of BS168 growth at different NaOx concentrations by a Kruskal-Wallis test with Dunn’s multiple comparisons. Only the comparison pairs shown had significantly different effects on BS168 growth. *, *P < *0.05; **, *P < *0.01; ***, *P < *0.001; ****, *P* < 0.0001; ns, not significant.

Since oxalate can be toxic to bacteria, even those capable of degrading it, the viability of BS168 upon exposure to oxalate concentrations ranging from 50 μM to 50 mM was assessed (18). Representative growth curves are presented in Fig. 1A. There was a significant, dose-dependent increase in growth when BS168 was supplemented with sodium oxalate (NaOx) (Fig. 1B to D).

### BS168 decreases stone burden and improves survival in a D. melanogaster model of urolithiasis.

**FIG 2:**
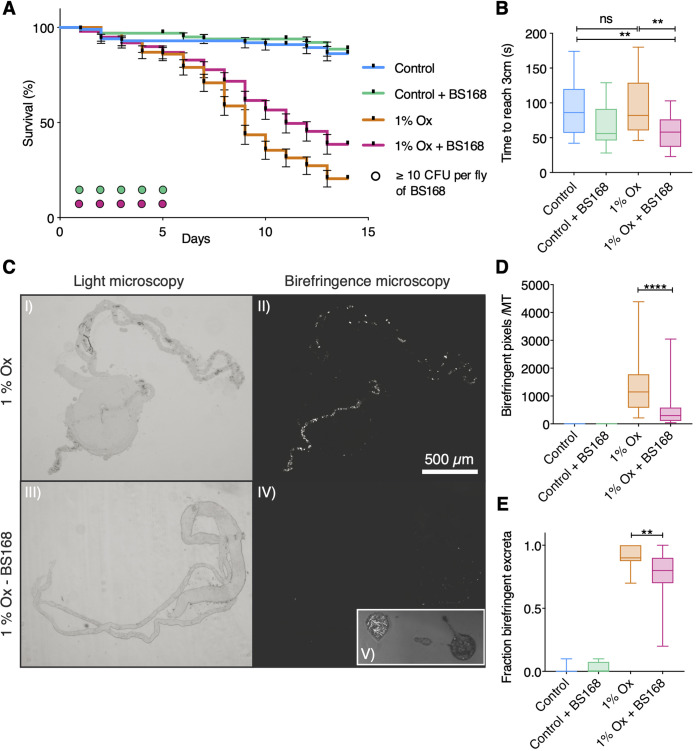
BS168 decreases stone burden and improves survival in a D. melanogaster model of urolithiasis. (A) Kaplan Meier survival analysis of 5-day old D. melanogaster adults that were supplemented with 2 × 10^8^ CFU of BS168 on day 0, transferred to normal or lithogenic medium on day 1, and followed for 14 days. Error bars represent standard errors (*n*, ≥100 per group from four biological replicates of 20 to 30 flies). BS168 at ≥10 CFU per fly was cultured on LB agar from pulverized BS168-supplemented D. melanogaster flies until experimental day 5, illustrated as circles colored by treatment group (*n*, 10 per group from three biological replicates). (B) Larval locomotion was determined on day 4 after larvae were supplemented with 5 × 10^7^ CFU of BS168 on day 0 and transferred to normal or lithogenic medium on day 2 (*n*, 19 to 24 larvae per group from three biological replicates). CaOx crystals were imaged in dissected Malpighian tubules (MT) from D. melanogaster adults on day 7 (C [I to IV]) and quantitated with ImageJ particle analysis (D) (*n*, 21 to 29 adults per group from three biological replicates). Birefringent fecal excreta from coverslips in adult vials on day 14 were imaged (C [V]), and the fraction that contained birefringent crystals was quantitated (E) (*n*, 16 to 20 coverslips per group from three biological replicates). The scale bar in panel C is relevant for panels I to IV. All box plots illustrate the median, quartiles, minimum, and maximum. **, *P < *0.01; ****, *P* < 0.0001; ns, not significant.

Stone burden and health were assayed in the D. melanogaster model of CaOx monohydrate urolithiasis (19, 20) after dietary supplementation with NaOx and BS168; adult survival, adult Malpighian tubule crystal birefringence, adult fecal excreta birefringence, and larval locomotion were all evaluated (Fig. 2). BS168 was detected by culture on Luria-Bertani (LB) agar from pulverized D. melanogaster adults up to 5 days following supplementation (Fig. 2A). Kaplan-Meier survival analysis of D. melanogaster adults (Fig. 2A) demonstrated that the detrimental effects of the highly lithogenic 1% NaOx diet were ameliorated with BS168 supplementation (*P*, 0.0057 by log rank test). Larval crawling was significantly increased in 4-day-old larvae treated with BS168 on lithogenic medium over that of untreated controls (data were parametric by a D’Agostino-Pearson test; *P*, 0.0073 for comparison with normal-medium controls by two-way analysis of variance [ANOVA] with Tukey’s multiple comparisons; *P*, 0.0013 for comparison with lithogenic controls by two-way ANOVA with Tukey’s multiple comparisons) (Fig. 2B). On day 7, dissected Malpighian tubules (Fig. 2C) from BS168-treated D. melanogaster adults had significantly less CaOx crystal deposition than those from untreated lithogenic controls (data were nonparametric by a D’Agostino-Pearson test; *P*, <0.0001 by a Wilcoxon rank sum test) (Fig. 2D). The percentage of fecal excreta containing birefringent particles from D. melanogaster adult vials after 14 days was significantly lower in the BS168-treated cohorts than in untreated lithogenic controls (data were nonparametric by a D’Agostino-Pearson test; *P*, 0.0039 by a Wilcoxon rank sum test) (Fig. 2E).

### BS168 rescues lithogenic-diet-mediated microbiota alterations in D. melanogaster.

The microbial composition of whole pulverized D. melanogaster adults was assessed after 7 days on normal or lithogenic medium following BS168 treatment on day 0. The common D. melanogaster endosymbiont genus *Wolbachia* dominated the sequencing depth of all fly samples (average relative abundance, 97.7%) but did not differ significantly between treatment cohorts (21). Although *Wolbachia* is known to impact the microbiota, fertility, viral infection susceptibility, and longevity, among other traits, in D. melanogaster, this bacterium is not known to play a role in the D. melanogaster urolithiasis model, so reads corresponding to the genus were removed from downstream analysis (21–24).

After the removal of sequencing control samples (based on their distinct clustering apart from D. melanogaster samples) (see Fig. S1 in the supplemental material), the D. melanogaster microbiota data set contained 1,974,659 total reads, ranging from 10,945 to 76,733 reads across the 40 samples. An average of 2.72% of reads were removed from each sample following quality filtration, performed utilizing the DADA2 pipeline (25). The remaining filtered 1,922,688 reads were assigned taxonomy (26). After filtering of sequence variants to maintain those present at >1% in any sample, 66 variants remained.

10.1128/mSphere.00498-20.2 FIG S1 Sequencing controls distinctly separate from D. melanogaster microbiota. Principal component analysis (PCA) plot of sequencing control and adult D. melanogaster samples. A PCA was performed on CLR-transformed Aitchison distances. Distance between samples on the plot represents differences in microbial community composition, with 34.9% of total variance being explained by the first two components shown. D. melanogaster samples are colored by treatment groups, and sequencing control samples are red. The black ellipse represents the 95% confidence interval of the D. melanogaster samples. Sequence variants are depicted by the gray numbers. Download FIG S1, JPG file, 0.6 MB. Copyright © 2020 Al et al. 2020 Al et al. This content is distributed under the terms of the Creative Commons Attribution 4.0 International license.

In agreement with past surveys of the D. melanogaster microbiota, flies consuming normal medium exhibited a distinct and low-diversity microbiota dominated by *Lactobacillus* and *Acetobacter* species (27). In the lithogenic-diet cohorts, the relative proportion of sequence variant 127, which, based on sequence homology, likely corresponds to Acetobacter tropicalis, was significantly lower than that in normal-medium controls (*P*, 0.0075 by a Benjamini-Hochberg-corrected Wilcoxon rank sum test), while lactobacilli proportions were unchanged. There were no differences in the abundances of any bacterial groups between BS168-treated and untreated groups. No sequences corresponding to the genus *Bacillus* were detected from the flies in any cohort.

**FIG 3:**
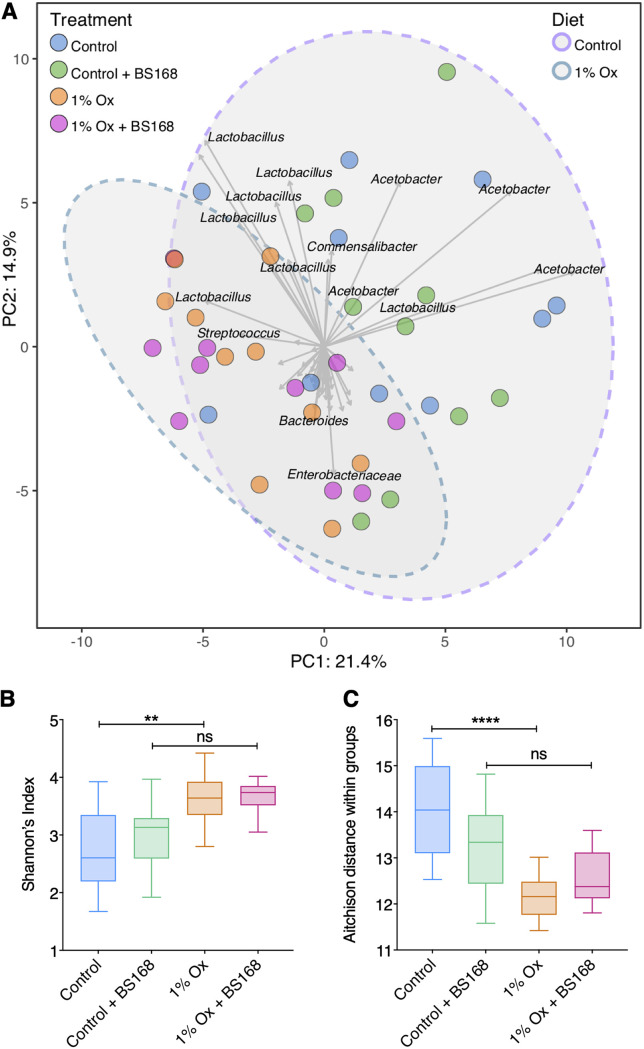
Exploratory analysis of D. melanogaster microbiota. (A) PCA plot of D. melanogaster adults exposed to lithogenic medium and BS168 supplementation. PCA was performed on CLR-transformed Aitchison distances. The distance between samples (colored points) on the plot represents differences in microbial community composition, with 36.3% of total variance being explained by the first two components shown. Strength and association for genera (sequence variants) are represented by the length and direction of the gray arrows, respectively. Individual samples are colored by treatment groups, and ellipses represent the 95% confidence intervals of the diet groups. (B) Shannon’s index of alpha diversity was calculated for each individual sample and plotted by treatment group. (C) The Aitchison distance within treatment groups was determined. The distance of every individual sample from all others within the same treatment group was averaged to obtain a single distance value per sample. In panels B and C, box plots illustrate the median, quartiles, and 5% to 95% confidence intervals. **, *P < *0.01; ****, *P < *0.0001; ns, not significant.

The sequence counts were centered log ratio (CLR) transformed, generating sample-wise Aitchison distances, which were subsequently used to perform a principal-component analysis (PCA) (Fig. 3A). Principal components 1 and 2 were plotted and represent 36.3% of the total variance in the data (Fig. 3A). Samples did not partition into distinct groups based on treatment (colored points); however, subtle drivers in the data separation were noted for diet groups and denoted with 95% confidence ellipses. Differences in diversity metrics (Shannon’s index of alpha diversity [Fig. 3B] and Aitchison distance determination [Fig. 3C]) due to exposure to the lithogenic diet were mitigated when D. melanogaster were supplemented with BS168.

**FIG 4:**
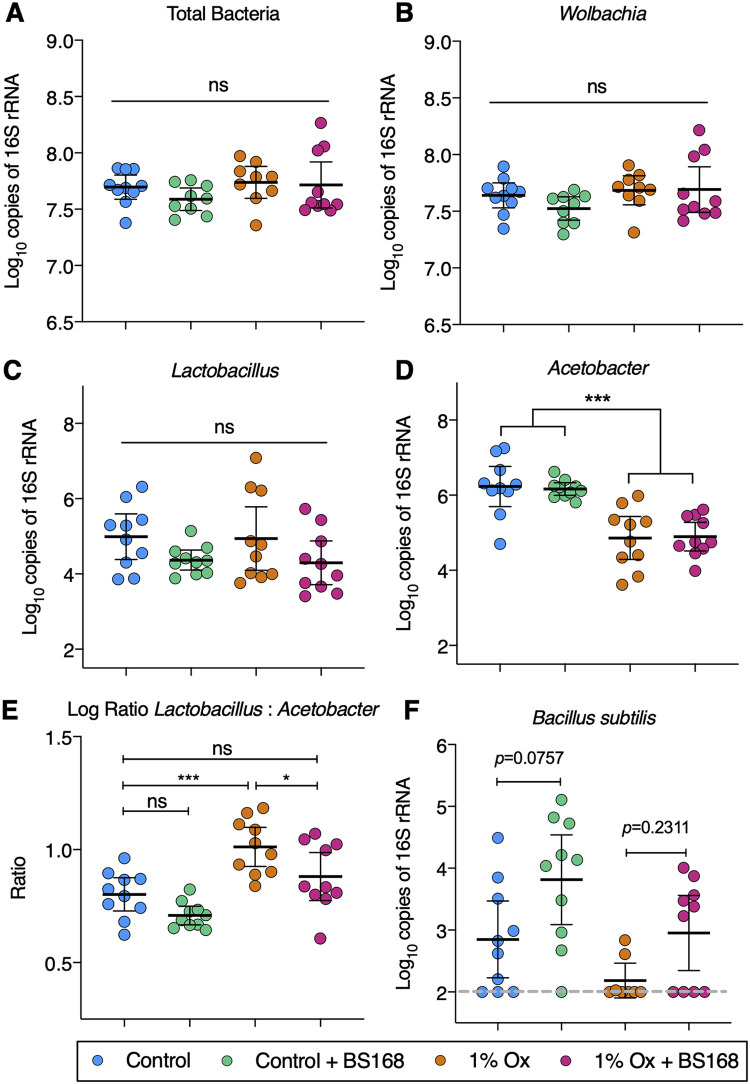
qPCR-based assessment of D. melanogaster microbiota. (A to F) Molecular quantification of total bacteria, bacterial genera, and species in whole-body D. melanogaster adults. (E) Intraindividual *Lactobacillus*/*Acetobacter* load ratios. All comparisons were made after normalizing to total host DNA. Data are depicted as means ± standard deviations. Significance was determined with Tukey’s multiple-comparison tests. Each point represents a single D. melanogaster adult from a separate experimental vial (*n *= 10; three technical replicates were performed). Sexes are pooled. *, *P < *0.05; ***, *P < *0.001; ns, not significant.

In addition to the 16S rRNA gene sequencing, qPCR of bacterial groups of interest was undertaken to determine total bacterial loads (Fig. 4). Total bacterial and *Wolbachia* sp. loads were unchanged between treatment groups (Fig. 4A and B, respectively). The abundance of the genus *Lactobacillus* did not differ significantly between groups; however, abundance of the genus *Acetobacter* was significantly decreased in the lithogenic-diet groups (Fig. 4C and D, respectively). The intraindividual ratio of *Lactobacillus* to *Acetobacter* was increased by the lithogenic diet (*P = *0.0007); however, this phenomenon was rescued with BS168 supplementation (the group given 1% NaOx plus BS168 was not significantly different from untreated controls; *P*, 0.048 between the group receiving 1% NaOx alone and the group receiving 1% NaOx plus BS168) (Fig. 4E). Species-specific primers were utilized to assess the load of B. subtilis; BS168-treated groups trended toward increased loads (Fig. 4F).

### Pretreatment with BS168 prevents increased calcium oxalate crystal aggregation and adhesion to renal epithelial cells.

**FIG 5:**
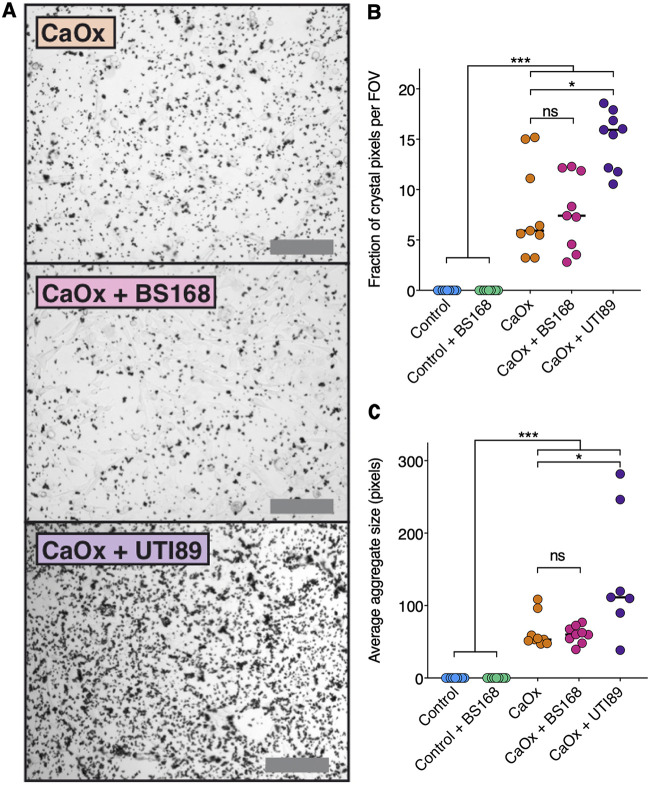
Pretreatment with BS168 prevents increased calcium oxalate crystal aggregation and adhesion to renal epithelial cells. (A) Crystal aggregates were visualized by light microscopy after MDCK monolayers were pretreated with MEM with or without BS168 or UTI89 (positive control), followed by artificial urine with or without 5 mg/ml CaOx monohydrate. Bars, 200 μm. (B) The amounts of CaOx monohydrate crystals adhering to MDCK cells did not differ significantly between the CaOx groups with or without BS168 pretreatment. (C) Average crystal aggregate sizes did not differ significantly between the CaOx groups with or without BS168 pretreatment. Each point represents the average from three fields of view (FOV) in a single well of a 6-well plate, and the median for each group is indicated by a horizontal line. Three technical and three biological replicates were performed (*n *= 9). Significance was determined by a Kruskal-Wallis test with Dunn’s multiple comparisons (*, *P < *0.05; ***, *P < *0.001; ns, not significant).

MDCK renal epithelial cells were utilized to assess the effect of BS168 pretreatment on the adhesion and aggregation of CaOx monohydrate crystals in artificial urine (Fig. 5A). Cells that were pretreated with BS168 prior to CaOx exhibited no more crystal adhesion than vehicle control cells, in contrast to cells that were pretreated with uropathogenic Escherichia coli (UPEC) strain UTI89, which was utilized as a positive control (Fig. 5B). Average crystal sizes did not differ significantly between the CaOx groups with or without BS168 pretreatment, indicating that BS168 did not encourage aggregation (Fig. 5C).

## DISCUSSION

This study is the first to show that B. subtilis 168 (BS168) can functionally reduce lithogenesis in an established D. melanogaster model of urolithiasis. Notably, BS168 gained a dose-dependent growth advantage in response to oxalate and, when used as an oral supplement for D. melanogaster on a lithogenic diet, could significantly reduce stone burden in both the Malpighian tubules and fecal excreta. These antilithogenic effects of BS168 were found to be independent of the D. melanogaster developmental stage and also extended to mitigating lithogenic-diet-induced alterations in the D. melanogaster microbiota. Furthermore, pretreatment with BS168 increased the survival of D. melanogaster adults during lethal exposure to oxalate, improved locomotor function in D. melanogaster larvae, and prevented increased adhesion and aggregation of CaOx crystals to renal epithelial cells *in vitro*. Collectively, these findings suggest that BS168 can mitigate the severity of urolithiasis *in vivo* and may hold promise as a future microbial therapeutic for human CaOx nephrolithiasis.

Oxalate is a toxin consumed in the diet and produced endogenously in the liver (5). Humans are incapable of degrading oxalate and instead rely on excretion and microbial degradation (8); accordingly, it is a common component in kidney stones (1). Many species of bacteria are able to degrade the compound; however, oxalate can still exert toxicity over bacterial cells, and many gut commensals cannot tolerate high levels of oxalate (18). Here, we demonstrated that BS168 exhibited prolific growth in medium with as much as 50 mM NaOx present, which is expected to far exceed physiological relevance based on an approximated 2.0 mM daily dietary oxalate ingestion (3). Along with its ability to form endospores, resist bile salts, and tolerate low-pH conditions, these results suggest that BS168 would likely survive well in the human intestinal tract despite even the highest levels of dietary oxalate consumption (28). Hatch and colleagues (29) demonstrated that the gut commensal bacterium O. formigenes not only degrades oxalate but also promotes enteric oxalate excretion from circulation. Future studies on BS168 should investigate this potential and evaluate the degree to which BS168 may lower serum and urinary oxalate concentrations.

The well-established D. melanogaster model of urolithiasis was utilized as a high-throughput screening tool to assess the beneficial properties of BS168 *in vivo* (19). This model is limited in that it is driven solely by dietary oxalate consumption (exceeding human physiological levels), which is not representative of the more-complex and multifactorial cascade that leads to stone formation in humans (19, 30). Additionally, the intracellular endosymbiont genus *Wolbachia* was prevalent in D. melanogaster here, but its relative abundance was equivalent across cohorts, mitigating differential outcomes based on colonization status (21). Finally, although the D. melanogaster microbiota is not representative of that of humans, its low-diversity structure enables the deconvolution of complexity when assessing the microbiota as an output metric following the lithogenic diet and bacterial treatments (31).

It was found that BS168 transiently colonized the intestinal tract for as long as 5 days following a single treatment, yet it could elicit marked improvements to D. melanogaster survival during at least 14 days of lethal oxalate exposure (19, 32, 33). These findings suggest that, in addition to directly metabolizing oxalate, BS168 may reduce oxalate toxicity indirectly through priming of host cell physiology (14). It is well known that renal oxalate toxicity is mediated primarily via mitochondrial dysfunction and excessive reactive oxygen species (ROS) generation induced by phospholipase A2 (PLA_2_) activation (34). This process leads to inflammation and damage to the renal epithelium, which can become a crystal deposition site, accelerating stone formation (35–37). Notably, B. subtilis can biosynthesize lipopeptides that are potent inhibitors of PLA_2_ and have been demonstrated *in vivo* to decrease inflammation (38, 39). This suggests that the survival benefits afforded by BS168 in oxalate-exposed D. melanogaster may be partly due to the prevention of oxalate-induced mitochondrial dysfunction via blunting of the PLA_2_-facilitated ROS signaling cascade.

In corroboration of these findings, BS168 increased psychomotor activity, indicative of improved metabolic energy conversion and neuronal development, in both oxalate-exposed and nonexposed D. melanogaster larvae (40–42). Given the integral role of PLA_2_ in modulating oxidative-stress-related degenerative diseases in D. melanogaster, these results indicate that BS168-mediated modulation of PLA_2_ activity may represent a key mechanism of indirect protection against oxalate-based stone disease (43). Recent evidence from studies investigating alternative mechanisms has also shown that B. subtilis from fermented foods can decrease mitochondrial dysfunction, oxidative stress, and DNA damage associated with metabolic dysfunction (44–46). Future studies should look to characterize the exact mechanism of action of BS168, but given the links between metabolic syndrome and urolithiasis, the conserved and multipronged ability of B. subtilis strains to promote mitochondrial health may offer a simple and effective solution for attenuating oxalate-induced renal damage in patients with recurrent stone formation (46, 47).

Oxalate has been shown to alter the microbiota in humans; however, how this impacts nephrolithiasis is still unclear (8, 18). For this reason, the simplicity of the D. melanogaster urolithiasis model was advantageous for evaluating the effect of the lithogenic diet on the microbiota. Indeed, it was found that oxalate consumption could exert significant effects on the D. melanogaster microbiota, some of which were mitigated by supplementation with BS168. Specifically, consumption of the lithogenic diet by D. melanogaster adults led to significant alterations in microbial alpha and beta diversity and, additionally, altered the ratio of the two dominant bacterial genera of the D. melanogaster microbiota, *Lactobacillus* and *Acetobacter*. In both instances, BS168 was able to rescue these phenotypes. These findings suggest that BS168 exerts its protective effects without saturating the D. melanogaster microbiota. Supporting this, BS168 was undetectable by culture from D. melanogaster beyond experimental day 5, while on day 7, no sequence variants corresponding to *Bacillus* spp. were detected by 16S rRNA gene sequencing, and by qPCR, the B. subtilis loads were nearing the limits of detection and comparable between all groups. This is an important feature of B. subtilis, because the potential existed for this organism, as an oxalate-tolerant sporeformer, to overtake the endogenous microbiota in a manner reminiscent of Clostridium difficile infection in humans; however, this was not observed (48).

It is unlikely that a large quantity of BS168 would be present in the kidney after oral administration, even though the gut is a reservoir for the urogenital microbiota (49). However, since gut colonization with uropathogenic Escherichia coli (UPEC) has been shown to increase the risk of UPEC urinary tract infections (UTI), experiments were performed here to address how BS168 may impact the urinary tract and stone development should some cells traffic there (50, 51). Previous studies have shown UPEC to aggregate on and around CaOx monohydrate crystals, significantly more than on other crystal compositions (52, 53). Other groups have shown the ability of CaOx monohydrate to adhere to MDCK renal epithelial cells, but the role of bacteria in this process had not been explored previously (54, 55). In the present study, BS168 did not encourage aggregation or adherence of CaOx to MDCK cells, in contrast to the UPEC strain UTI89, which acted as a positive control. Accordingly, these *in vitro* findings suggest that if any orally consumed BS168 cells did migrate to the urinary tract, increased morbidity would be unlikely.

In summary, this study characterized the beneficial properties of BS168 in the context of nephrolithiasis, as assayed in a well-established D. melanogaster model of the disease and *in vitro* cell culture experiments. Although probiotics are classically *Lactobacillus* or *Bifidobacterium* spp., strains of B. subtilis are generally regarded as safe, gaining favor as probiotics for gut-related maladies and are components of several fermented foods (16, 17, 56). To date, studies employing various formulations of probiotics in nephrolithiasis patients have largely been inconclusive due to the broad variety of preparations tested as well as ambiguous strain selection, making it unclear how efficacious this approach could be (12, 13, 57–60). Instead, future studies should carefully evaluate mechanistically validated strains that can be delivered effectively to the gut, such as BS168. Based on our seminal findings, this microbe may prove a novel therapeutic adjunct for reducing the incidence of recurrent CaOx nephrolithiasis in high-risk patients.

## MATERIALS AND METHODS

### Bacterial culture and growth curves.

Bacillus subtilis strain 168 (ATCC 23857) and Escherichia coli (UTI89) were routinely cultured at 37°C in Luria-Bertani (LB) broth (61). Growth in oxalate was assayed in 96-well plates prepared as dilutions of sodium oxalate (NaOx) in LB broth with stationary-phase bacteria added at a final dilution of 1/100. Plates were incubated for 24 h at 37°C with optical density (OD) readings every 30 min using an Eon microplate spectrophotometer (BioTek, Winooski, VT, USA). NaOx concentrations were selected based on physiologic intestinal oxalate concentrations reported in the literature (62). Growth curves were analyzed with the R package Growthcurver and GraphPad Prism (version 8.1.2) (63).

D. melanogaster flies were cultured following homogenization until day 10 of the survival assay to assess BS168 loads. D. melanogaster flies were surface sterilized with 70% ethanol and homogenized in 0.01 M phosphate-buffered saline (PBS) by using a motorized pestle. Homogenates were diluted up to 100-fold, plated onto LB agar, and then incubated aerobically at 37°C for 24 h. The characteristic colony morphology of BS168 was easily differentiated on LB, which is not a growth medium amenable to culturing the typical D. melanogaster microbiota (64).

### Drosophila melanogaster husbandry.

Wild-type Canton-S (stock no. 1) flies were obtained from the Bloomington *Drosophila* Stock Center at Indiana University. D. melanogaster flies were maintained using a medium with 1.5% (wt/vol) agar, 1.73% (wt/vol) yeast (catalog no. 51475; Sigma-Aldrich), 7.3% (wt/vol) cornmeal, 7.6% (vol/vol) corn syrup, and 0.58% (vol/vol) propionic acid at 25°C with 12-h light-dark cycles. The lithogenic diet included 1.0% (wt/vol) NaOx, which was added prior to medium solidification. All adult D. melanogaster experiments were performed in wide polypropylene vials (model GEN32-121; Diamed Lab Supplies, Inc., Mississauga, Ontario, Canada) containing 10 ml of medium, and where specified, larval experiments were performed in polypropylene fly bottles (model GEN32-130; Diamed) containing 50 ml of medium. Life span measurement was performed as described previously (65).

### Determining the effect of BS168 in a D. melanogaster stone model.

An experimental timeline is displayed in Fig. S2 in the supplemental material. At the age of 5 days, D. melanogaster adults were sorted into cohorts and were given a 5% sucrose solution as a supplement, with or without BS168, for 24 h. Bacteria were prepared for supplementation to D. melanogaster as follows: a 25-ml overnight broth culture of BS168 (∼10^8^ CFU/ml) was washed twice and resuspended in a 5% (wt/vol) sterile sucrose solution. D. melanogaster flies were transferred to polypropylene vials, each containing a cotton ball moistened with 3 ml of 5% sucrose with or without BS168. After 24 h, D. melanogaster flies were transferred to a standard medium with or without 1.0% NaOx.

10.1128/mSphere.00498-20.3 FIG S2 Drosophila melanogaster experimental timeline. (A) Schematic outline of experimental design. Adults were supplemented +/- BS168 in 3 ml of 5% sucrose via cotton balls in empty vials on Day 0. On day 1. they were transferred to standard medium vials with or without 1.0% NaOx (weight/volume). Deaths were recorded daily, and dead flies were removed every two days during transfer to fresh food. Fecal excreta were assayed throughout the 14-day experiment by transferring the coverslip-embedded vial plug with each food transfer. (B) D. melanogaster adults mated for 3 h in standard medium bottles, after which they were removed and 500 μl of 5% sucrose with or without BS168 was pipetted over the top of the medium. They were transferred via 20% sucrose suspension to standard medium with or without 1.0% NaOx on day 2, and their crawling was assayed on day 4. Download FIG S2, JPG file, 0.6 MB. Copyright © 2020 Al et al. 2020 Al et al. This content is distributed under the terms of the Creative Commons Attribution 4.0 International license.

For larval exposure to BS168, on day 0 approximately 200 D. melanogaster adults were mated in standard medium bottles for 3 h. Five hundred microliters of 5% sucrose with or without BS168 (processed as described above) was pipetted on top of the medium after adults were removed. On day 2, 100 ml of a room temperature 20% sucrose solution was added to completely submerge the medium and float the larvae for 20 min. The sucrose solution containing larvae was then gently poured over a sterile cell strainer (model C431750; Corning, Oneonta, NY, USA). The larvae collected in the strainer were briefly rinsed with 70% ethanol and then twice with deionized (DI) water. Cleaned larvae were added with a paint brush to vials of standard medium with or without 1.0% NaOx.

Stone burden was evaluated in adult D. melanogaster Malpighian tubules on day 7. Briefly, D. melanogaster flies were narcotized with CO_2_, treated for 2 min in Carl’s solution (66) in a small glass petri dish, dissected in cold PBS using a Nikon SMZ800N stereomicroscope, fixed in 4% formaldehyde-PBS for 1 h at room temperature, and mounted on microscope slides in PBS and glycerol (50:50). Clear nail polish was used to seal the coverslip to the microscope slide prior to polarized light microscopy. Birefringence microscopy of the dissected tubules was performed with a Nikon Ts2R inverted microscope, and NIH ImageJ software was utilized to determine the degree of birefringence per tubule (particle analysis function). Fecal excreta from D. melanogaster adults were also evaluated for the presence of birefringent crystals throughout the duration of the 14-day survival analysis, as described previously (32).

Stone burden was evaluated in third-instar D. melanogaster larvae on day 4 by means of a crawling assay; larval crawling is an indicator of behavioral and locomotor health in D. melanogaster (67, 68). Twenty microliters of a room temperature 20% sucrose solution was added to each medium bottle to float larvae for 20 min. The solution was poured over a sterile cell strainer, where the collected larvae were washed twice with DI water. A paint brush was used to transfer larvae from the strainer to a 15-ml petri dish containing 2% agar, where they acclimated for 10 min. A second petri dish of 2% agar was positioned over a 0.5-cm graph paper grid. A 6-mm-diameter paper disk was submerged in apple cider vinegar and positioned near one side of the plate, and larvae were positioned at a starting point 4 cm away. The time it took each larva to travel 3 cm from the starting point toward the vinegar stimulant was recorded.

### CaOx crystal adhesion to renal tissue culture.

Cell culture experiments were performed in MDCK renal epithelial cells (54, 55, 69), which were acquired from the American Type Culture Collection (ATCC CCL-34). Cells were maintained in T75 flasks in a 5% CO_2_ tissue culture incubator with minimum essential medium supplemented with 10% fetal bovine serum and 2 mM l-glutamine, the combination of which is referred to below as MEM. For experiments, cells were seeded in 6-well tissue culture treated plates at 1 × 10^5^/ml and were incubated until confluence at approximately 48 h.

An overnight broth culture of BS168 and UTI89 was processed as described above, but the bacterial pellet was reconstituted in MEM at a concentration of ∼5 × 10^3^ CFU/ml. MDCK monolayers were washed twice with PBS, incubated with 2 ml MEM with or without BS168 or UTI89 for 20 min, washed twice with PBS again, and then incubated with 2 ml of artificial urine with or without 0.5 mg/ml CaOx monohydrate for 20 min (70). The urine was removed, and cells were washed twice with PBS; then 2 ml MEM was added to each well, and cells were immediately imaged by light microscopy using a Nikon Ts2R inverted microscope. Crystal attachment was quantitated with ImageJ.

Statistical analysis for D. melanogaster and cell culture experiments was conducted with GraphPad Prism. Results were considered significant as follows: ****, *P* < 0.0001; ***, *P* < 0.001; **, *P* < 0.01; *, *P* < 0.05.

### 16S rRNA gene sequencing.

Analysis of the adult D. melanogaster microbiota was carried out by 16S rRNA gene sequencing of 10 individual flies per cohort on day 7. Specifically, five sex-separated vials were prepared per treatment group containing approximately 20 flies, and 1 fly (either male or female) was used from each. DNA was extracted from the single whole flies in accordance with the Earth Microbiome Project standard protocols, using the Qiagen DNeasy PowerSoil 96-well kit (Qiagen, Toronto, Ontario, Canada). A Biomek 3000 laboratory automation workstation was utilized for PCR reagent setup. Amplifications of the V4 region of the 16S rRNA gene were carried out as described previously (71). Processing of DNA samples and DNA sequencing were conducted at the London Regional Genomics Centre at Robarts Research Institute (London, Ontario, Canada). Amplicons were quantified using PicoGreen and were pooled at equimolar concentrations before cleanup (QIAquick PCR cleanup; Qiagen, Germantown, MD). The final samples were sequenced using the MiSeq by Illumina platform, with 2 × 260-bp paired-end chemistry. The returned reads were then analyzed using R, DADA2, the SILVA database (version 132), and ALDEx2 (25, 26, 72, 73).

### qPCR-based quantification of microbial communities in D. melanogaster.

The DNA template from the Qiagen DNeasy PowerSoil kit was also utilized for qPCR-based quantification. Bacterial loads were determined by qPCR using the Power SYBR green kit (Applied Biosystems) according to the manufacturer’s instructions. The universal 16S rRNA gene-, genus-, and species-specific primer sets used in this study are listed in Table S1 (74–78). All qPCRs were performed in DNase- and RNase-free 384-well microplates on a Quant Studio 5 real-time PCR system (Applied Biosystems) and were analyzed with associated software. Copy numbers of target 16S rRNA genes were calculated as described previously using established primer efficiencies and limits of detection (79, 80).

10.1128/mSphere.00498-20.4 TABLE S1 Primers used for qPCR. Download Table S1, PDF file, 0.1 MB. Copyright © 2020 Al et al. 2020 Al et al. This content is distributed under the terms of the Creative Commons Attribution 4.0 International license.

### Data availability.

All 16S rRNA gene sequencing data have been deposited in the NCBI Sequence Read Archive under BioProject ID no. PRJNA634107.
