# Supplementary figures and images for: Oxalate-Degrading Bacillus subtilis Mitigates Urolithiasis in a Drosophila melanogaster Model
Source: mSphere. 2020 Sep 9;5(5):e00498-20. doi: 10.1128/mSphere.00498-20 (PMC7485683; doi:10.1128/mSphere.00498-20)

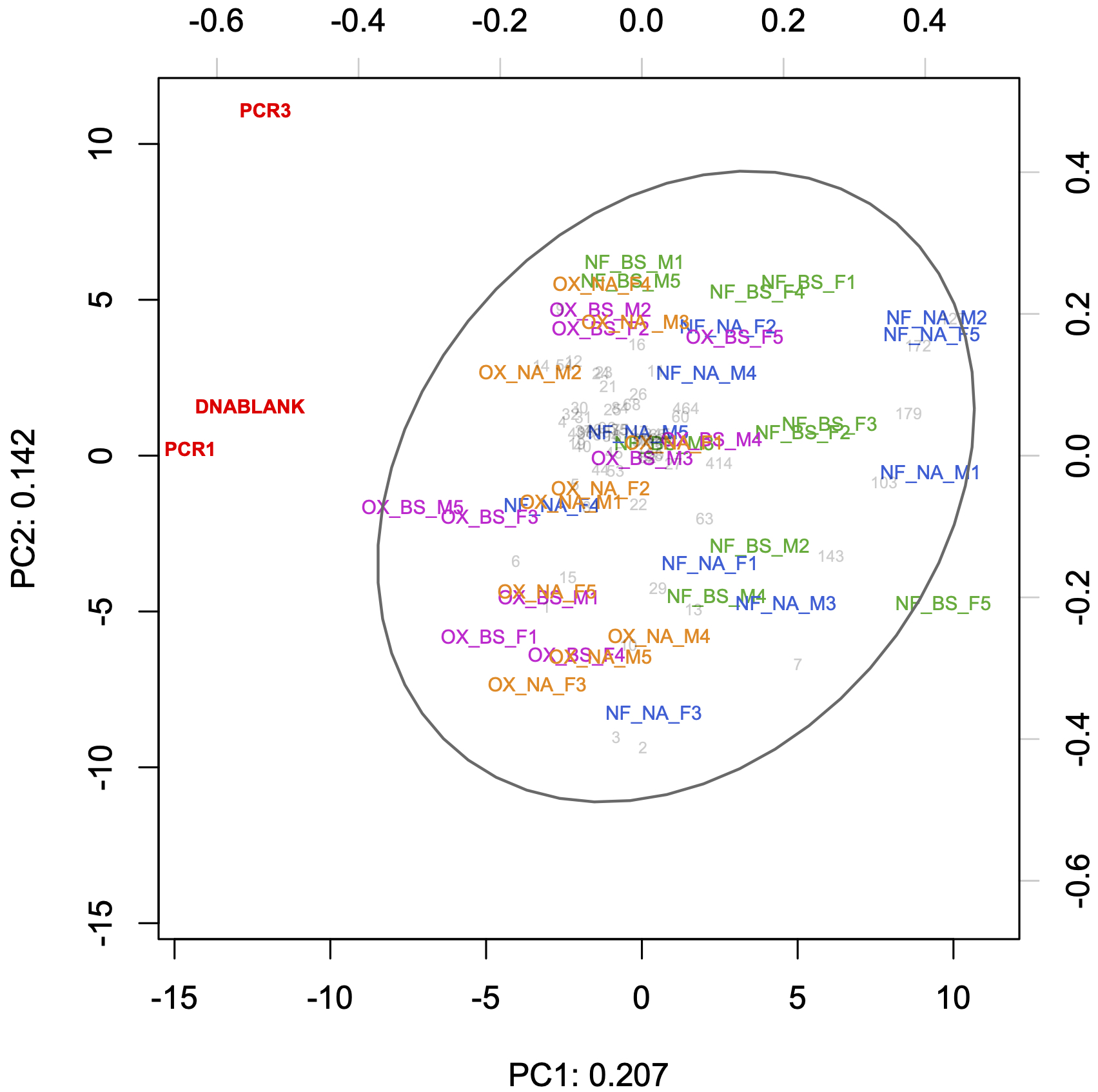

Supplement: FIG S1 [file mSphere.00498-20-sf001.jpg]

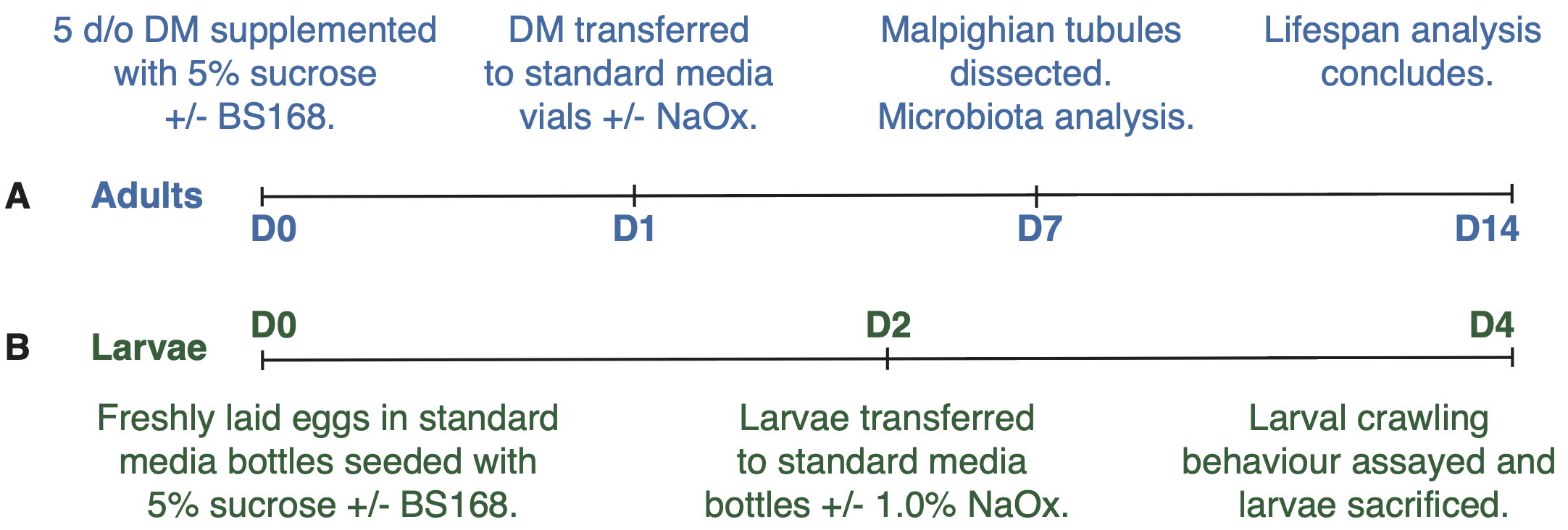

Supplement: FIG S2 [file mSphere.00498-20-sf002.jpg]
