# Supplementary material for: Oxalate-Degrading Bacillus subtilis Mitigates Urolithiasis in a Drosophila melanogaster Model
Source: mSphere. 2020 Sep 9;5(5):e00498-20. doi: 10.1128/mSphere.00498-20 (PMC7485683; doi:10.1128/mSphere.00498-20)
Supplement: TABLE S1 [file mSphere.00498-20-st001.pdf]

**Target**	**Primer**	**Sequence (5’>3’)**	**Reference**
Host endogenous control	DRO_B-act_F	GGAAACCACGCAAATTCTCAGT	(1)
	DRO_B-act_R	CGACAACCAGAGCAGCAACTT	
Host endogenous control	DRO_RpLP0_F	CCGAAAAGTCTGTGCTTTGTTCT	(2)
	DRO_RpLP0_R	CGCTGCCTTGTTCTCCCTAA	
Universal bacteria	BAC_UNI_F	ACTCCTACGGGAGGCAGCAGT	(3)
	BAC_UNI_R	ATTACCGCGGCTGCTGGC	
Acetobacter spp.	BAC_Aceto_F	TAGTGGCGGACGGGTGAGTA	(1)
	BAC_Aceto_R	AATCAAACGCAGGCTCCTCC	
Lactobacillus spp.	BAC_Lacto_F	AGGTAACGGCTCACCATGGC	(1)
	BAC_Lacto_R	ATTCCCTACTGCTGCCTCCC	
Wolbachia spp.	BAC_wsp_F	CATTGGTGTTGGTGTTGGTG	(4)
	BAC_wsp_R	ACCGAAATAACGAGCTCCAG	
Bacillus subtilis	BAC_B-subtilis_F	GCGGCGTGCCTAATACATGC	(5)
	BAC_B-subtilis_R	CTCAGGTCGGCTACGCATCG	

REFERENCES

1. Elgart M, Stern S, Salton O, Gnainsky Y, Heifetz Y, Soen Y. 2016. Impact of gut microbiota on the fly's germ line. Nat Commun 7:11280.
2. Daisley BA, Trinder M, McDowell TW, Welle H, Dube JS, Ali SN, Leong HS, Sumarah MW, Reid G. 2017. Neonicotinoid-induced pathogen susceptibility is mitigated by Lactobacillus plantarum immune stimulation in a Drosophila melanogaster model. Sci Rep 7:2703.
3. Hartman AL, Lough DM, Barupal DK, Fiehn O, Fishbein T, Zasloff M, Eisen JA. 2009. Human gut microbiome adopts an alternative state following small bowel transplantation. Proc Natl Acad Sci U S A 106:17187-92.
4. Newton IL, Savytskyy O, Sheehan KB. 2015. Wolbachia utilize host actin for efficient maternal transmission in Drosophila melanogaster. PLoS Pathog 11:e1004798.
5. Lahlali R, Peng G, Gossen BD, McGregor L, Yu FQ, Hynes RK, Hwang SF, McDonald MR, Boyetchko SM. 2013. Evidence that the biofungicide Serenade (Bacillus subtilis) suppresses clubroot on canola via antibiosis and induced host resistance. Phytopathology 103:245-54.
